# Significance of APOB/APOA1 Ratio in the Prediction of Calcific Aortic Valve Disease

**DOI:** 10.1155/cdr/5528174

**Published:** 2025-08-03

**Authors:** Yuxing Wang, Ming Yu, Song Yang, Jiajie Mei, Zhenzhu Liu, Zhaohong Geng, Wenli Xie, Lijiao Zhang, Hongyan Wang, Nan Niu, Peng Qu

**Affiliations:** Department of Cardiology, The Second Hospital of Dalian Medical University, Dalian, Liaoning, China

**Keywords:** APOB/APOA1, calcific aortic valve disease, prediction model

## Abstract

**Background:** Calcific aortic valve disease (CAVD) is a prevalent heart valve disease. The ratio of two apolipoproteins with distinct functions, Apolipoprotein B/Apolipoprotein A1 (APOB/APOA1), has been proposed as a novel assessment index for the evaluation of cardiovascular diseases. The aim of this article is to discuss the role of lipid parameters such as APOB/APOA1 in CAVD and the risk factors for CAVD, to develop a predictive model for CAVD, and to evaluate the sensitivity and specificity of this model.

**Method:** Patients who initially presented to the Department of Cardiology of the Second Affiliated Hospital of Dalian Medical University between 1 January 2023 and 31 December 2023 were retrospectively identified and included in the study. Patients were divided into an aortic valve calcification group (111 cases) and a control group (201 cases) based on computed tomography (CT) findings. The clinical data, laboratory examination results, and chest CT images of the patients were collected and analyzed. A variety of statistical methods were used to analyze risk factors for CAVD, to construct a CAVD prediction model, and to assess its sensitivity and specificity.

**Results:** Lipid parameters APOA1, APOB/APOA1, cumulative low-density lipoprotein (LDL) exposure, and non–high-density lipoprotein/high-density lipoprotein (non-HDL/HDL) were significantly associated with aortic valve calcification. Age, history of diabetes, diastolic blood pressure (DBP), APOB/APOA1, Cystatin C (Cys-c), and neutrophil-to-lymphocyte ratio (NLR) are identified as independent risk factors for CAVD, and the combined model achieved an AUC of 0.796 for CAVD prediction, corresponding to a sensitivity of 0.769 and a specificity of 0.755.

**Conclusion:** The lipid parameters APOA1, APOB/APOA1, cumulative LDL exposure, and non-HDL/HDL have been demonstrated to be associated with aortic valve calcification. Furthermore, APOB/APOA1 can be used for the prediction of CAVD, and the combination of APOB/APOA1 with age, history of diabetes, DBP, Cys-c, and NLR has better prediction performance for CAVD.

## 1. Introduction

CAVD is a prevalent form of valvular heart disease, ranking as the third most common cardiovascular disorder after coronary heart disease and hypertension [1]. According to the Global Burden of Disease Study 2019 [2], which assessed trends in the global burden of cardiovascular disease from 1990 to 2019, it shows that heart valve disease accounts for about 2.5% of deaths from cardiovascular disease of different causes, and that globally, the prevalence and age-standardized prevalence of CAVD have risen steadily in the last three decades; it has risen from approximately 4.6 to 11.6 cases per 100,000 people and is clinically important because ventricular outflow tract obstruction due to severe calcification can lead to left ventricular dysfunction requiring surgical or transcatheter valve replacement [3], which poses a serious personal and global economic and medical burden.

The early presentation of CAVD is atherosclerosis of the aortic valve leaflets, with a prevalence of calcification or sclerosis of the aortic valve of 20%–30% in individuals over the age of 65 and 48% in those over the age of 85 [4]. Severe calcification of the aortic valve in advanced stages results in the development of aortic stenosis, which in turn leads to obstruction of the left ventricular outflow tract. This, in turn, leads to heart failure, for which surgical valve replacement is the only effective treatment. Liu Li [5] found that the prevalence of valvular calcification was 13.4% in a random sample of the elderly population in Beijing, 7.7% in those aged 60 years or older, 16.1% in those aged 70 years or older, and 25.7% in those aged 80 to 89 years.

The notion that CAVD is merely a passive degenerative change associated with age has long been a widely held view. Recent studies [6] have provided new insights into the pathogenesis of calcific aortic stenosis, indicating that it is an active progressive disease influenced by multiple risk factors. Epidemiological studies [7, 8] have demonstrated a strong correlation among age, dyslipidemia, and diabetes mellitus and the development of calcific aortic stenosis. Histopathological studies [9–14] have revealed that calcific aortic stenosis involves inflammation, abnormal lipid metabolism, matrix remodeling, and calcification.

CAVD shares numerous similarities with atherosclerosis, both in terms of risk factors and pathological changes. Indeed, it has been postulated that CAVD represents an additional manifestation of atherosclerosis [15]. Lipids play a significant role in the pathogenesis of CAVD. While the majority of lipid management guidelines identify LDL as the causative lipid component and have demonstrated that LDL reduction can reduce the risk of cardiovascular events, attempts have been made to block or slow the progression of CAVD with statin therapy. The results of a study [16] in animal models have indicated that hypercholesterolemia can result in the development of aortic valve sclerosis and hemodynamic changes. Additionally, a randomized clinical trial [17] has suggested that lowering blood lipids may potentially slow the progression of valve calcification and stenosis; however, despite these findings, large-scale, prospective, controlled trials have not consistently demonstrated that statins are effective in ameliorating the progression and progression of calcific aortic stenosis [18–21]. Thus, the role of lipids in CAVD is controversial, and other components of lipids may also be involved in the formation of CAVD.

Previous studies usually focused on traditional indicators such as LDL, but they reflected only the promoting effects of atherosclerosis, and the effects of each component of the lipid profile on atherosclerosis were different, whereas APOB/APOA1, as a novel lipid parameter, took into account both promoting and inhibiting effects of atherosclerosis in the lipid profile and better reflected the trend of the effects of the lipid profile on atherosclerosis. APOB was recognized as a risk-predicting biomarker in a large sample size study [22], which revealed that APOB is responsible for the transportation of a vast array of potentially atherogenic cholesterol, including very LDL, intermediate-density lipoprotein, and LDL; conversely, HDL is transported by APOA1 [23]. In the context of various lipid abnormalities, a multitude of factors influence the balance between proatherogenic and antiatherogenic lipoprotein particles. The most utilized indicators in the clinical assessment of lipids encompass total cholesterol (TC), triglyceride (TG), LDL, and HDL. The management of dyslipidemia continues to present significant challenges, as residual cardiovascular risk persists even after LDL levels attain a target standard. This suggests that other components of lipids, in addition to LDL, may also play a role in the development of cardiovascular disease [24, 25]. Searching for new lipid parameters associated with cardiovascular risk may help prevent disease, and therefore, a series of nontraditional lipid parameters have been derived from lipids, including cumulative exposure to LDL [26], APOB/APOA1, non-HDL, residual cholesterol, and atherogenic index of plasma (AIP) [27], which are all important factors in the development of cardiovascular disease.

Therefore, the objective of this study is threefold: firstly, to examine the risk factors associated with CAVD; secondly, to investigate the correlation between lipids and their derived parameters (such as LDL and APOB/APOA1 ratio) and CAVD; and thirdly, to construct a multifactorial prediction model including APOB/APOA1 and evaluate its clinical application value.

## 2. Methods

### 2.1. Study Design

This is a retrospective cohort study, in which patients who first visited the Department of Cardiology of the Second Hospital of Dalian Medical University between 1 January 2023 and 31 December 2023 are being retrospectively collected. All patients underwent a comprehensive chest CT scan upon admission. We defined aortic valve calcification as CT values ≥ 130 Hu in the aortic valve region, and 312 patients were ultimately included in the study after the exclusion of individuals with comorbidities such as pregnancy, severe hepatic and renal insufficiency, hematological diseases, tumors, autoimmune disorders, inflammatory diseases, hyperthyroidism, hypothyroidism, rheumatic heart disease, cardiomyopathy, dilated cardiomyopathy, and myocarditis. Independently predicted long-term oral lipid-lowering medications, such as statins, were excluded.

Statistical simulation study [28] showed that according to the method of event per variable (EPV), the general EPV is at least 10–15. With the final inclusion of six independent variables in this study, each independent variable should have 60–90 patients with CAVD, and the incidence of CAVD is about 30%, so the number of inclusion is 200–300. The number of sample cases included in this study was 312, which meets the sample size requirement.

Agatston established a minimum threshold of CT for calcified areas at 130 Hu, defining the area with CT values exceeding this threshold as calcified. This was further divided into 111 cases in the calcified group and 201 cases in the control group based on the presence or absence of aortic valve calcification in CT. The general information of the two groups was also collected, including gender. The general data, including gender, age, height, weight, body mass index (BMI), body surface area (BSA), history of hypertension, history of diabetes, and history of coronary heart disease, were collected from the two groups. The results of blood tests, including those pertaining to lipids, were collated from the patients. Thereafter, lipid-derived parameters were calculated, along with the Agatston calcification score (details are shown in Table 1). The correlation between aortic valve calcification and lipids and their derived parameters was investigated by comparing the differences in lipids and their derived parameters between the two groups. A CAVD prediction model was constructed using Spearman's correlation analysis, binary logistic regression, and the ROC curve, and the model was evaluated for sensitivity and specificity. This study was approved by the Ethics Committee of the Second Affiliated Hospital of Dalian Medical University and exempt from signing an informed consent form (Ethics Number KY2025-110-01).

### 2.2. Statistical Analyses

The statistical analysis of the obtained data was conducted using the SPSS 26.0 software package. The count data were expressed by a constitutive ratio, and a *X*^2^ test was employed. The measurement data were tested for normality using a S-W test and a Q-Q plot. If they conformed to normality, they were expressed by the mean ± standard deviation (*X* ± SD). If the data did not conform to normality, they were expressed by M (Q25, Q75), and the nonparametric test was used. If the data conformed to normal distribution and met the chi-square, an independent sample *t*-test analysis was conducted. If the data met the normal distribution but did not meet the chi-square, an *t*′ − test was performed. The correlation between aortic valve calcification and factors was analyzed using Spearman's correlation analysis and one-way logistic regression analysis. Binary multifactorial regression analysis (stepwise method) was performed for factors with *p* value less than 0.05. Indicators with *p* value less than 0.05 were included in the analyses and plotted on a ROC graph. Forest plots were drawn using R software to visualize CVD risk factors. Calibration curves were plotted using R software and Hollingshead–Luce tests were performed to assess the validity of the predictive models. A statistically significant difference was observed when *p* value is less than 0.05.

## 3. Results

**Figure 1:**
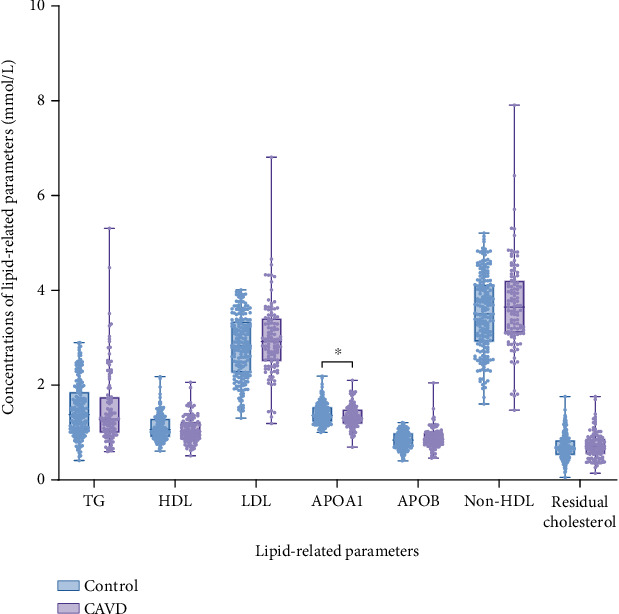
Box plots comparing each general lipid parameter in the CAVD group and the control group. Note: ⁣^∗^*p* < 0.05, ⁣^∗∗^*p* < 0.01; compared to the control group, the difference was statistically significant.

**Figure 2:**
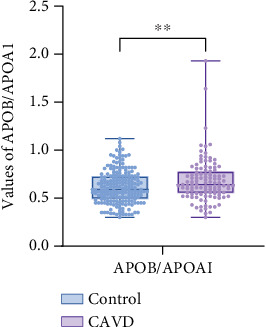
Box plots comparing APOB/APOA1 in the CAVD group and the control group. Note: ⁣^∗^*p* < 0.05,^∗∗^*p* < 0.01; compared to the control group, the difference was statistically significant.

**Figure 3:**
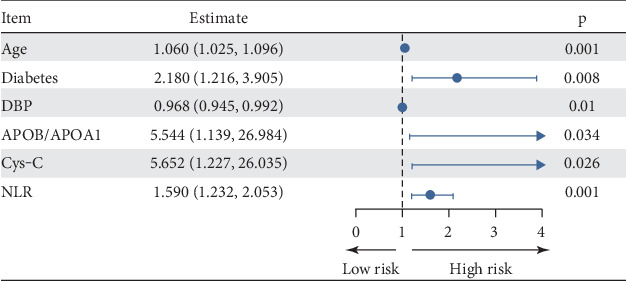
Forest map of CAVD risk factors. Note: DBP, diastolic blood pressure; APOB/APOA1, Apolipoprotein B/Apolipoprotein A1; Cys-c, cystatin C; NLR, neutrophil-to-lymphocyte ratio.

**Figure 4:**
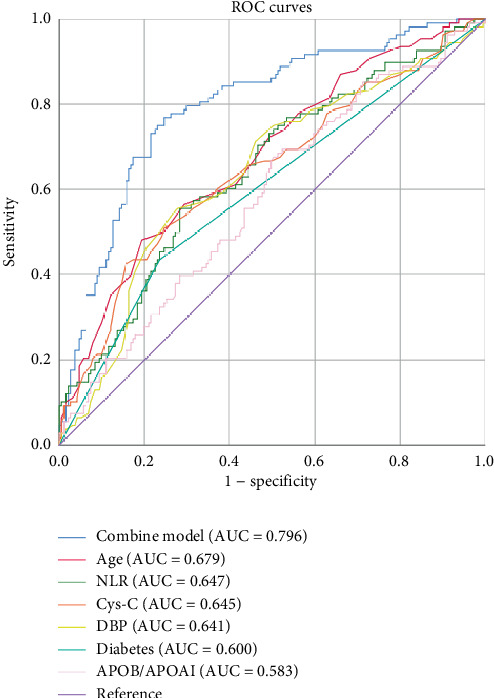
ROC curves for aortic valve calcification and various risk factors.

**Figure 5:**
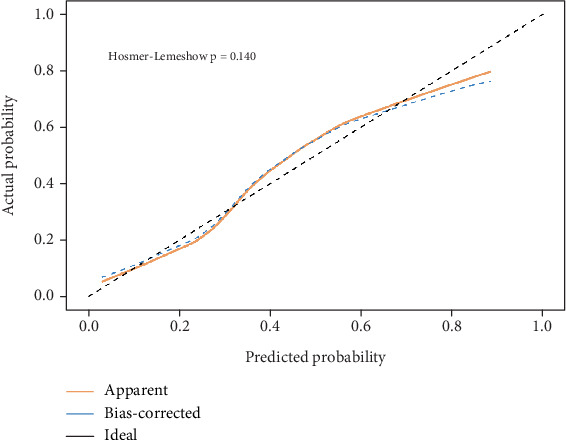
CAVD prediction model calibration curve. Note: Apparent, calibration curve for predicted incidence; bias-corrected, measured incidence curve; ideal, ideal reference line.

1. The difference in age, history of diabetes mellitus, and DBP was statistically significant in the calcification group compared to the control group, while the difference in smoking, alcohol consumption, history of hypertension, history of coronary heart disease, height, weight, BMI, BSA, and systolic blood pressure (SBP) was not statistically significant (details are shown in Table 2).
2. Compared to the control group, the differences in APOA1 (1.34 ± 0.24 vs. 1.40 ± 0.22), APOB/APOA1 (0.68 ± 0.23 vs. 0.62 ± 0.17), cumulative exposure to LDL (201.29 ± 55.00 vs. 171.63 ± 49.05), and non-HDL/HDL (3.71 ± 1.48 vs. 3.37 ± 1.119) were statistically significant in patients in the calcification group. In addition, the differences in blood glucose, glycated hemoglobin A1c (HbA1c), urea, Cys-c, sodium, chlorine, calcium, neutrophil percentage (NEUT%), lymphocyte percentage (LY%), NLR, and d-dimer were statistically significant (details are shown in Table 3 and Figures 1 and 2).
3. Spearman correlation results showed that aortic valve calcification was correlated with age, history of coronary heart disease, history of diabetes mellitus, DBP, blood glucose, HbA1c, cumulative LDL exposure, APOA1, APOB/APOA1, non-HDL/HDL, Cys-c, LDH, sodium, chlorine, calcium, NEUT%, LY%, NLR, and d-dimer.
  The Agatston score for aortic valve calcification was correlated with age, history of coronary artery disease, history of diabetes mellitus, DBP, blood glucose, HbA1c, cumulative LDL exposure, non-HDL/HDL, APOB/APOA1, urea, Cys-c, LDH, sodium, chlorine, NEUT%, LY%, NLR, and D-dimer (details are shown in Table 4).
4. The results of univariate logistic regression showed that aortic valve calcification was associated with age, history of diabetes mellitus, DBP, blood glucose, HbA1c, cumulative LDL exposure, APOA1, APOB/APOA1, non-HDL/HDL, urea, Cys-c, sodium, chloride, calcium, NEUT%, LY%, NLR, and D-dimer (details are shown in Table 5).
5. The factors associated with aortic valve calcification were subjected to binary logistic regression; whether before or after the exclusion of confounding factors, the results demonstrated that age, history of diabetes mellitus, DBP, APOB/APOA1, Cys-c, and NLR were independent risk factors for aortic valve calcification (details are shown in Table 6 and Figure 3).
6. The ROC analysis included the variables age, history of diabetes, DBP, APOB/APOA1, Cys-c, and NLR. The areas under the curve for these variables were 0.679, 0.600, 0.641, 0.583, 0.645, and 0.647; the area under the curve for the combined prediction of aortic valve calcification by the above indexes was 0.796, and the maximum Uden index of this prediction model was 0.522, corresponding to a sensitivity of 0.769 and a specificity of 0.754 (details are shown in Table 7 and Figure 4).
7. The results of the calibration curve show that the calibration curve of the predicted incidence rate and the measured incidence rate curve are both close to the ideal reference line, and the *p* value of the H-L test is greater than 0.05, which indicates that there is no significant difference between the model and the expected model, suggesting that the efficacy of the model is good (details are shown in Figure 5).

## 4. Discussion

Globally, there is a clear transition in the incidence of cardiovascular disease from the young to the old, with an exponential increase with age [29]. CAVD is a common cardiovascular disease, and a large-scale survey demonstrated that the prevalence of aortic stenosis was approximately 0.4% in individuals younger than 45 years of age, 1.5% in those aged 65 years and older, and 3.4% in those aged 75 years and older [30]. As in previous studies, the results of the present study showed that the mean age of the aortic valve calcification group was significantly higher than that of the control group, suggesting an increase in the occurrence of CAVD with increasing age.

Studies have clearly classified CAVD pathology into lipid deposition (early stage), inflammatory fibrosis (middle stage), and calcification (late stage), with the rate of progression positively correlating with the baseline lipid level and the number of risk factors, such as hypertension and diabetes mellitus. The rabbit model on a high cholesterol diet showed that lipid deposition in the valve appeared within 3 months, the inflammatory response intensified within 6–12 months, and calcified plaque formed within 18–24 months, which corresponds to 15–20 years in humans when scaled to the life span of the species [6]. A study explored the relationship between LDL and aortic valve calcification by integrating data from multiple community-based cohorts, which included a total of 6942 patients, and found that for every 1 mmol/L increase in LDL, there was a 28% increase in the risk of developing CAVD [31]. But a study analyzing valve tissue from 102 patients with aortic stenosis found that an elevated proportion of small, dense LDL particles was significantly associated with intervalvular oxidative LDL deposition, inflammatory response, and stenosis progression, even when total LDL levels were normal. Thus, single LDL is not a good predictor of aortic valve disease [32]. It has also been suggested that an elevated APOB/APOA1 ratio in patients with metabolic syndrome independently predicts the risk of aortic valve calcification, even when LDL is normal [33], which suggested the combination of APOB/APOA1 with hypertension, diabetes, and hyperlipidemia is a valid predictor of CAVD.

Prior research has demonstrated that the APOB/APOA1 is a risk factor for cardiovascular disease and is associated with an unfavorable prognosis for cardiovascular disease [34, 35]. APOB and APOA1 levels are predictors of cardiovascular events and all-cause mortality in patients with chronic kidney disease [36]. APOB/APOA1, as a novel lipid parameter, shows a unique clinical value in the field of cardiovascular disease. The APOB/APOA1 has significant advantages over traditional lipid markers, and its core value is reflected in the accurate reflection of atherosclerosis mechanisms and the improvement of clinical predictive efficacy. APOB, as the main apolipoprotein of atherogenic lipoproteins such as LDL, intermediate-density lipoprotein, and very low-density lipoprotein, directly corresponds to the number of atherogenic particles in the circulation, whereas APOA1 is the core component of HDL that plays an antiatherosclerotic role. APOB/APOA1 can assess the pathophysiological status of lipoprotein metabolism in a dynamic and balanced manner, which compensates for the limitation that LDL only reflects cholesterol content and ignores particle heterogeneity [37]. A change in any of these indices leads to an imbalance in the APOB/APOA1 [38, 39].

A comprehensive exploration of the underlying mechanisms and their application to clinical practice is of paramount importance in enhancing the early recognition and diagnosis of cardiovascular diseases, thus providing a novel direction for the diagnosis and treatment of cardiovascular diseases.

In this study, we found APOB/APOA1 can be used for the prediction of CAVD. APOB acts as a ligand for the surface receptor of LDL, transports cholesterol from the liver to the periphery, and induces platelet activation, degranulation, and adhesion release to promote the inflammatory response; alternatively, natural polymorphic APOB danger-associated Signaling 1 has been found to efficiently activate platelets and promote platelet–leukocyte interactions, which plays an important role in the promotion of inflammatory response by APOB [40]. APOA1 is the main protein component in HDL, which can inhibit platelet activation, reduce clot strength and stability by inhibiting thromboxane A2 release, and bind with HDL receptor, which not only promotes reverse cholesterol transport and prevents cholesterol from being deposited abnormally and damaging the vascular endothelium but also activates the activity of inducible nitric oxide synthase, thus maintaining endothelial cell integrity and acting as a protective agent [41, 42]. APOB drives inflammatory activation via nuclear factor kappa-light-chain-enhancer of activated B-cell (NF-*κ*B) signaling, whereas APOA1 exerts protective effects through ATP-binding cassette transporter A1 (ABCA1)–dependent cholesterol efflux and nod-like receptor family pyrin domain Containing 3 (NLRP3) inflammasome inhibition [43–46]. While the NLR ratio reflects the level of systemic inflammation, the APOB/APOA1 and NLR were significantly higher in the aortic valve calcification group than in the control group, which suggests that the APOB/APOA1 is an indicator of the balance between lipid and inflammatory responses in patients with aortic valve calcification. A high APOB/APOA1 suggests that the balance between “promotion” and “inhibition” is disrupted, which may explain the increased risk of aortic valve calcification with an elevated APOB/APOA1.

Moreover, the results of a study encompassing 17,745 participants, with a follow-up period of 19.8 years, indicated that an increase of 30% in the APOB/APOA1 ratio was associated with a 30% rise in the risk of aortic valve stenosis [47]. A study of 159 patients with aortic stenosis, followed over a period of 2 years, revealed that stenosis progressed at a rate of 3.4 times faster in patients with APOB/APOA1 ratios ≥ 0.62 than in those with low ratios [48]. Some studies have confirmed the general risk prediction value of APOB/APOA1 in global populations and the superiority of APOB/APOA1 over HDL or LDL assays alone for early risk identification, which provides strong support for early risk identification in cardiovascular disease [49]. The findings of the present study demonstrated that while the area under the ROC curve (AUC = 0.583) of a solitary APOB/APOA1 indicator was comparatively constrained (see Table 7), its correlation with CAVD is still better than other lipid parameters, such as HDL and LDL. In addition, the effectiveness of its amalgamated model with other indicators was satisfactory for the prediction of CAVD (AUC of the amalgamated model = 0.796, sensitivity = 0.769, and specificity = 0.754). The results of a randomized, double-blind, placebo-controlled trial [50] demonstrated that an abnormal APOB/APOA1 (> 0.7) significantly improved the predictive power of traditional cardiovascular disease risk models [51, 52] and the results of our study are similar to those of this study. In patients with CAVD, an elevated APOB/APOA1 may promote inflammation via lipid deposition and osteogenic differentiation of the VIC through the modulation of pathways such as the Wnt pathway [47].

These findings support the use of the APOB/APOA1 in combination with other risk indicators in clinical practice to assess CAVD risk, especially in populations where traditional indicators are insufficient for a comprehensive assessment of risk, and whereby clinicians can stratify their patients and develop personalized preventive programs, such as lifestyle modification and initiation of pharmacological interventions, to reduce the incidence of cardiovascular events.

Despite the encouraging application prospects of the APOB/APOA1, further study is required on this front. Firstly, large-scale, multicenter prospective studies should be conducted to clarify the optimal threshold values of the APOB/APOA1 in different populations (different ages, genders, and races), so as to improve its accuracy and usefulness in clinical diagnosis. Secondly, the combination of the APOB/APOA1 with other parameters should be explored to further improve its predictive ability for CAVD risk [53]. In addition, combining multiomics studies to explore the specific signaling pathways and key factors involved in the APOB/APOA1 affecting valve calcification will help to investigate therapeutic agents targeting these pathways and key factors, thereby slowing down the progression of CAVD.

This study also found that CAVD is associated with the following factors: diabetes, blood pressure, and Cys-c.

The prevalence of diabetes is increasing year by year, and there are now more than 150 million people with diabetes globally. Diabetes is associated with the development of several cardiovascular diseases. Diabetes mellitus is also associated with vascular calcification, which has a complex pathological mechanism involving synergistic effects of metabolic disorders, inflammatory responses, and abnormal cellular functions at multiple levels [54, 55]. Wang et al.'s study identified diabetes mellitus as risk factors for calcific heart valve disease through the analysis of their medical history [56]. Cheng's study finds that diabetes is associated with the development of degenerative heart valve disease [57]. In conjunction with the results of this study, it is illustrated that diabetes mellitus is positively associated with calcific aortic valve lesions and is a risk factor for the development of calcific aortic valve lesions.

Blood pressure is strongly associated with the development and prognosis of many cardiovascular diseases [58, 59]. A Mendelian randomization study [60] showed that both diastolic and systolic blood pressures were significantly associated with several cardiovascular diseases, including myocardial infarction, increasing the risk of these diseases. Zhang et al.'s study found that low DBP is a risk factor for diastolic insufficiency of the heart [61]. Similar results were found in the study by Chen et al. [62]. In terms of pathological changes, a decrease in DBP results in a slowing of blood flow at the aortic valve, increasing the likelihood of stagnation. This promotes contact between blood components and the aortic valve, allowing for reactions with the valve. This results in the promotion of aortic valve calcification, which in turn leads to incomplete valve closure and the regurgitation of some ventricular blood during diastole. This further contributes to the reduction in DBP. The results of Iwata et al.'s study indicated that DBP was negatively correlated with aortic valve calcification [63]. In conjunction with the results of this study, DBP was negatively correlated with CAVD, suggesting that elevated DBP inhibits aortic valve calcification.

Cys-c is a class of low molecular weight nonglycosylated proteins and a member of the human cysteine protease inhibitor superfamily [64]. Cys-c is widely distributed in human tissue cells and blood, and the kidney is the only metabolic pathway for Cys-c, which is filtered in the glomerulus and reabsorbed and catabolized in the proximal tubule. Some studies have confirmed that Cys-c is more accurate and sensitive to the early and slight changes in glomerular filtration rate and can be used to assess the early stage of renal function impairment [65]. In recent years, Cys-c has been found to be valuable in the prediction of cardiovascular disease, with one study suggesting that Cys-c is independently associated with coronary artery calcification [66]. Elevated Cys-c is associated with coronary atherosclerotic plaque formation in Vakili et al.'s study [67]. Cho et al.'s study finds Cys-c to be a valid marker for predicting cardiovascular disease progression or new onset [68]. Cys-c may induce osteoblast-like cell differentiation and promote vascular calcification by upregulating the expression of osteogenesis-related genes (Runx2 and osteocalcin) [69]. In this study, Cys-c was found to be positively associated with CAVD and is a risk factor for aortic valve calcification.

In conclusion, this study clarifies the correlation between lipids and cardiovascular disease, although the predictive value of a single APOB/APOA1 indicator for CAVD is relatively limited, but still statistically significant; it correlates better with CAVD than traditional lipid parameters, and the APOB/APOA1 ratio together with age, history of diabetes mellitus, DBP, Cys-c, and NLR constitutes a good predictive efficacy for CAVD in a combined model.

## 5. Conclusions

The lipid parameters APOA1, APOB/APOA1, cumulative LDL exposure, and non-HDL/HDL have been demonstrated to be associated with aortic valve calcification. Furthermore, APOB/APOA1 can be used for the prediction of CAVD, and the combination of APOB/APOA1 with age, history of diabetes, DBP, Cys-c, and NLR has better prediction performance for CAVD.

### 5.1. Limitations and Prospects

It must be acknowledged that the present study is a single-center, small-sample, retrospective study, which may have resulted in the observed results being affected by other vascular calcifications. Consequently, it would be beneficial for future multicenter, large sample, prospective, follow-up studies to be conducted in order to confirm these findings.

## Figures and Tables

**Table 1:** Calculation method of Agatston calcification score.

**A** **g** **a** **t** **s** **t** **o** **n** **calcification** **score** = ∑ **area** **of** **calcification** **in** **each** **CT** **cross** − **section**∗**coefficient** **corresponding** **tothemaximum** **CT** **value** **ofthe** **calcified** **plaque**	**Maximum CT value**	**Modulus**
Calculate the area of calcification in each cross-section Calcification score of the cross − section = plaque area∗coefficient corresponding to maximum CT value Agatston calcification score = sum of calcification integrals for each cross-section	130–199	1
	200–299	2
	300–399	3
	≥ 400	4

**Table 2:** Comparison of baseline information between patients in CAVD and control groups.

**Item**	**Calcification group** **N** = 111	**Control group** **N** = 201	**Test value (** **t**/**t**′/**X**^2^**)**	**p**
Male (*n*, %)	(70, 63.1%)	(109, 54.2%)	2.282	0.131
Smoking (*n*, %)	(44, 39.6%)	(88, 43.8%)	0.502	0.478
Drinking (*n*, %)	(21, 18.9%)	(33, 16.4%)	0.313	0.576
Hypertensive (*n*, %)	(76, 68.5%)	(125, 62.2%)	1.230	0.267
Diabetes (*n*, %)	(49, 44.1%)	(48, 24.0%)⁣^∗∗∗^	13.497	< 0.001
Coronary heart disease (*n*, %)	(67, 60.4%)	(100, 49.8%)	3.236	0.072
Age (year)	68.09 ± 9.20	60.97 ± 11.06^∗∗∗^	−5.773	< 0.001
Height (m)	1.66 ± 0.13	1.67 ± 0.08	0.414	0.679
Weight (kg)	72.04 ± 13.97	73.23 ± 13.41	0.741	0.459
BMI (kg/m^2^)	27.03 ± 14.04	26.27 ± 3.83	−0.718	0.473
BSA (m^2^)	1.78 ± 0.22	1.80 ± 0.21	0.735	0.463
SBP (mmHg)	136.69 ± 20.05	138.55 ± 18.64	0.819	0.414
DBP (mmHg)	81.59 ± 12.47	86.68 ± 11.73^∗∗∗^	3.591	< 0.001

Abbreviations: BMI, body mass index; BSA, body surface area; DBP, diastolic blood pressure; SBP, systolic blood pressure.

⁣^∗^*p* < 0.05, ⁣^∗∗^*p* < 0.01, ⁣^∗∗∗^*p* < 0.001: statistically significant difference.

**Table 3:** Comparison of laboratory findings between patients in CAVD and control groups.

**Item**	**Calcification group** **N** = 111	**Control group** **N** = 201	**Test value (** **t**/**t**′/**X**^2^**)**	**p**
Blood glucose (mmol/L)	6.66 ± 2.72	5.88 ± 1.71^∗∗^	−2.739	0.007
HbA1c (mmol/L)	6.82 ± 1.66	6.24 ± 1.22^∗∗^	−3.243	0.001
TC (mmol/L)	4.76 ± 0.95	4.63 ± 0.80	−1.244	0.215
TG (mmol/L)	1.51 ± 0.81	1.49 ± 0.57	−0.328	0.743
HDL (mmol/L)	1.07 ± 0.28	1.11 ± 0.27	1.458	0.146
LDL (mmol/L)	2.97 ± 0.77	2.82 ± 0.66	−1.771	0.078
Cumulative LDL exposure (mmol/L·year)	201.29 ± 55.00	171.63 ± 49.05^∗∗∗^	−4.894	< 0.001
APOA1 (mmol/L)	1.34 ± 0.24	1.40 ± 0.22^∗^	2.042	0.042
APOB (mmol/L)	0.88 ± 0.22	0.84 ± 0.18	−1.914	0.057
APOB/APOA1	0.68 ± 0.23	0.62 ± 0.17^∗∗^	−2.878	0.004
Non-HDL (mmol/L)	3.69 ± 0.93	3.52 ± 0.80	−1.718	0.087
Non-HDL/HDL	3.71 ± 1.48	3.37 ± 1.19^∗^	−2.252	0.025
Residual cholesterol (mmol/L)	0.73 ± 0.29	0.70 ± 0.27	−0.815	0.416
AIP	0.12 ± 0.26	0.11 ± 0.24	−0.408	0.683
Urea (mmol/L)	6.11 ± 1.81	5.71 ± 1.37^∗^	−2.038	0.043
Creatinine (mmol/L)	69.53 ± 14.16	66.73 ± 14.62	−1.626	0.105
UA (mmol/L)	352.21 ± 88.47	339.83 ± 86.00	−1.205	0.229
Cys-c (mmol/L)	1.11 ± 0.23	1.01 ± 0.16^∗∗∗^	−4.274	< 0.001
ALP (U/L)	78.78 ± 26.79	75.73 ± 20.62	−1.120	0.263
LDH (mmol/L)	221.79 ± 146.11	196.39 ± 93.15	−1.654	0.100
Potassium (mmol/L)	3.97 ± 0.37	3.96 ± 0.31	−0.473	0.636
Sodium (mmol/L)	141.33 ± 2.99	142.09 ± 2.20^∗^	2.339	0.020
Chlorine (mmol/L)	106.32 ± 2.78	107.06 ± 2.14^∗^	2.429	0.016
Calcium (mmol/L)	2.22 ± 0.13	2.26 ± 0.12^∗^	2.599	0.010
WBC (×10^9^/L)	6.51 ± 1.88	6.46 ± 1.85	−0.196	0.845
NEUT%	66.01 ± 8.15	62.31 ± 8.59^∗∗∗^	−3.694	< 0.001
LY%	25.00 ± 7.43	29.15 ± 7.35^∗∗∗^	4.735	< 0.001
NLR	3.03 ± 1.47	2.36 ± 0.93^∗∗∗^	−4.906	< 0.001
d-Dimer (*μ*g/mL)	0.64 ± 0.31	0.57 ± 0.22^∗^	−2.488	0.013

Abbreviations: AIP, atherogenic index of plasma; ALP, alkaline phosphatase; APOA1, Apolipoprotein A1; APOB, Apolipoprotein B; Cys-c, Cystatin C; HbA1c, glycated hemoglobin A1c; HDL, high-density lipoprotein; LDH, lactate dehydrogenase; LDL, low-density lipoprotein; LY%, lymphocyte percentage; NEUT%, neutrophil percentage; NLR, neutrophil-to-lymphocyte ratio; non-HDL, non–high-density lipoprotein; TC, total cholesterol; TG, triglyceride; UA, uric acid; WBC, white blood cell.

⁣^∗^*p* < 0.05, ⁣^∗∗^*p* < 0.01, ⁣^∗∗∗^*p* < 0.001: statistically significant difference.

**Table 4:** Correlation analysis of aortic valve calcification and Agatston score with various factors.

**Item**	**Aortic valve calcification**		**Agatston score**	
	**Correlation coefficient**	**p**	**Correlation coefficient**	**p**
Sex	0.086	0.066	0.091	0.055
Age	0.313⁣^∗∗∗^	< 0.001	0.332⁣^∗∗∗^	< 0.001
Height	0.027	0.319	0.024	0.334
Weight	−0.013	0.407	−0.027	0.320
BMI	−0.037	0.259	−0.053	0.176
BSA	0.004	0.472	−0.006	0.457
Smoking	−0.040	0.240	−0.051	0.185
Drinking	0.032	0.289	0.038	0.255
Coronary heart disease	0.102⁣^∗^	0.036	0.110⁣^∗^	0.027
Hypertensive	0.063	0.134	0.057	0.160
Diabetes	0.208⁣^∗∗∗^	< 0.001	0.210⁣^∗∗∗^	< 0.001
SBP	−0.063	0.133	−0.042	0.228
DBP	−0.223⁣^∗∗∗^	< 0.001	−0.220⁣^∗∗∗^	< 0.001
Blood glucose	0.126⁣^∗^	0.013	0.111⁣^∗^	0.025
HbA1c	0.159⁣^∗∗^	0.003	0.154⁣^∗∗^	0.003
TC	0.048	0.199	0.060	0.145
TG	−0.064	0.131	−0.060	0.145
HDL	−0.084	0.068	−0.075	0.094
LDL	0.070	0.108	0.078	0.086
Cumulative LDL exposure	0.257⁣^∗∗∗^	< 0.001	0.275⁣^∗∗∗^	< 0.001
APOA1	−0.112⁣^∗^	0.024	−0.093	0.051
APOB	0.078	0.084	0.087	0.064
APOB/APOA1	0.139⁣^∗∗^	0.007	0.135⁣^∗∗^	0.009
Non-HDL	0.074	0.098	0.083	0.071
Non-HDL/HDL	0.114⁣^∗^	0.022	0.112⁣^∗^	0.024
Residual cholesterol	0.045	0.216	0.060	0.146
AIP	−0.003	0.478	−0.009	0.437
Urea	0.091	0.054	0.106⁣^∗^	0.031
Creatinine	0.090	0.056	0.082	0.075
UA	0.063	0.133	0.050	0.188
Cys-c	0.242⁣^∗∗∗^	< 0.001	0.234⁣^∗∗∗^	< 0.001
ALP	0.033	0.283	0.025	0.332
LDH	0.114⁣^∗^	0.022	0.107⁣^∗^	0.030
Potassium	0.013	0.408	0.031	0.295
Sodium	−0.101⁣^∗^	0.038	0.099⁣^∗^	0.041
Chlorine	−0.130⁣^∗^	0.011	−0.138⁣^∗∗^	0.008
Calcium	−0.113⁣^∗^	0.024	−0.088	0.062
WBC	0.007	0.453	−0.007	0.453
NEUT%	0.194⁣^∗∗∗^	< 0.001	0.218⁣^∗∗∗^	< 0.001
LY%	−0.250⁣^∗∗∗^	< 0.001	−0.268⁣^∗∗∗^	< 0.001
NLR	0.241⁣^∗∗∗^	< 0.001	0.262⁣^∗∗∗^	< 0.001
d-Dimer	0.100⁣^∗^	0.040	0.102⁣^∗^	0.037

Abbreviations: AIP, atherogenic index of plasma; ALP, alkaline phosphatase; APOA1, Apolipoprotein A1; APOB, Apolipoprotein B; BMI, body mass index; BSA, body surface area; Cys-c, Cystatin C; DBP, diastolic blood pressure; HbA1c, glycated hemoglobin A1c; HDL, high-density lipoprotein; LDH, lactate dehydrogenase; LDL, low-density lipoprotein; LY%, lymphocyte percentage; NEUT%, neutrophil percentage; NLR, neutrophil-to-lymphocyte ratio; non-HDL, non–high-density lipoprotein; SBP, systolic blood pressure; TC, total cholesterol; TG, triglyceride; UA, uric acid; WBC, white blood cell.

⁣^∗^*p* < 0.05, ⁣^∗∗^*p* < 0.01, ⁣^∗∗∗^*p* < 0.001: statistically significant difference.

**Table 5:** One-way regression analysis of aortic valve calcification with various factors.

**Item**	**B**	**p**	**OR**	**95% CI**	
				**Lower limits**	**Upper limits**
Sex	0.365	0.132	1.441	0.896	2.317
Age	0.074⁣^∗∗∗^	< 0.001	1.076	1.047	1.107
Height	−0.473	0.679	0.623	0.066	5.873
Weight	−0.006	0.458	0.994	0.977	1.010
BMI	0.009	0.495	1.009	0.983	1.036
BSA	−0.416	0.462	0.660	0.218	1.997
Smoking	−0.170	0.479	0.843	0.526	1.351
Drinking	0.172	0.576	1.188	0.649	2.173
CHD	0.430	0.073	1.538	0.961	2.461
Hypertensive	0.278	0.268	1.320	0.808	2.158
Diabetes	0.897⁣^∗∗∗^	< 0.001	2.452	1.496	4.018
SBP	−0.005	0.413	0.995	0.983	1.007
DBP	−0.036⁣^∗∗^	0.001	0.965	0.945	0.985
Blood glucose	0.164⁣^∗∗^	0.003	1.179	1.056	1.315
HbA1c	0.285⁣^∗∗^	0.001	1.330	1.125	1.573
TC	1.172	0.215	1.187	0.905	1.557
TG	0.058	0.742	1.060	0.749	1.502
HDL	−0.653	0.147	0.520	0.215	1.258
LDL	0.300	0.080	1.349	0.965	1.887
Cumulative LDL exposure	0.011⁣^∗∗∗^	< 0.001	1.011	1.006	1.017
APOA1	−1.085⁣^∗^	0.043	0.338	0.118	1.969
APOB	1.160	0.060	3.191	0.954	10.679
APOB/APOA1	1.749⁣^∗∗^	0.006	5.747	1.644	20.087
Non-HDL	0.239	0.089	1.270	0.965	1.671
Non-HDL/HDL	0.202⁣^∗^	0.029	1.224	1.021	1.466
Residual cholesterol	0.350	0.415	1.418	0.612	3.286
AIP	0.199	0.682	1.221	0.470	3.170
Urea	0.168⁣^∗^	0.030	1.182	1.017	1.375
Creatinine	0.013	0.106	1.013	0.997	1.029
UA	0.002	0.229	1.002	0.999	1.004
Cys-c	3.031⁣^∗∗∗^	< 0.001	20.720	5.118	83.877
ALP	0.006	0.265	1.006	0.996	1.016
LDH	0.002	0.081	1.002	1.000	1.004
Potassium	0.169	0.635	1.184	0.589	2.379
Sodium	−0.120⁣^∗^	0.014	0.887	0.806	0.976
Chlorine	−0.130⁣^∗^	0.011	0.878	0.795	0.970
Calcium	−2.656⁣^∗^	0.011	0.070	0.009	0.542
WBC	0.013	0.844	1.013	0.893	1.148
NEUT%	0.053⁣^∗∗∗^	< 0.001	1.054	1.024	1.086
LY%	−0.078⁣^∗∗∗^	< 0.001	0.925	0.893	0.957
NLR	0.503⁣^∗∗∗^	< 0.001	1.654	1.322	2.069
d-Dimer	1.132⁣^∗^	0.018	3.102	1.210	7.955

Abbreviations: AIP, atherogenic index of plasma; ALP, alkaline phosphatase; APOA1, Apolipoprotein A1; APOB, Apolipoprotein B; BMI, body mass index; BSA, body surface area; Cys-c, Cystatin C; DBP, diastolic blood pressure; HbA1c, glycated hemoglobin A1c; HDL, high-density lipoprotein; LDH, lactate dehydrogenase; LDL, low-density lipoprotein; LY%, lymphocyte percentage; NEUT%, neutrophil percentage; NLR, neutrophil-to-lymphocyte ratio; non-HDL, non–high-density lipoprotein; SBP, systolic blood pressure; TC, total cholesterol; TG, triglyceride; UA, uric acid; WBC, white blood cell.

⁣^∗^*p* < 0.05, ⁣^∗∗^*p* < 0.01, ⁣^∗∗∗^*p* < 0.001: statistically significant difference.

**Table 6:** Binary logistic regression analysis of aortic valve calcification.

**Item**	**Before excluding confounding factors**		**After excluding confounding factors**			
	**B**	**p**	**B**	**p**	**OR**	**95% CI**
Age	0.058	0.001⁣^∗∗^	0.058	0.001⁣^∗∗^	1.060	1.025–1.096
Diabetes	0.835	0.005⁣^∗∗^	0.779	0.008⁣^∗∗^	2.180	1.216–3.905
DBP	−0.034	0.007⁣^∗∗^	−0.032	0.010⁣^∗^	0.968	0.945–0.992
APOB/APOA1	1.827	0.026⁣^∗^	1.713	0.034⁣^∗^	5.544	1.139–26.984
Cys-c	1.673	0.032⁣^∗^	1.732	0.026⁣^∗^	5.652	1.227–26.035
NLR	0.464	< 0.001⁣^∗∗∗^	0.464	< 0.001⁣^∗∗∗^	1.590	1.232–2.053

Abbreviations: APOB/APOA1, Apolipoprotein B/Apolipoprotein A1; Cys-c, Cystatin C; DBP, diastolic blood pressure; NLR, neutrophil-to-lymphocyte ratio.

⁣^∗^*p* < 0.05, ⁣^∗∗^*p* < 0.01, ⁣^∗∗∗^*p* < 0.001: statistically significant difference.

**Table 7:** ROC curves for aortic valve calcification and various risk factors.

**Item**	**AUC**	**95% CI**		**p**
		**Lower limits**	**Upper limits**	
Combine model	0.796	0.743	0.850	< 0.001
Age	0.679	0.616	0.742	< 0.001
Diabetes	0.600	0.532	0.668	0.004
DBP	0.641	0.575	0.707	< 0.001
APOB/APOA1	0.583	0.516	0.649	0.018
Cys-c	0.645	0.578	0.712	< 0.001
NLR	0.647	0.582	0.712	< 0.001

*Note:* Statistically significant difference: *p* < 0.05.

Abbreviations: APOB/APOA1, Apolipoprotein B/Apolipoprotein A1; Cys-c, Cystatin C; DBP, diastolic blood pressure; NLR, neutrophil-to-lymphocyte ratio.

## Data Availability

The data that support the findings of this study are available from the corresponding author upon reasonable request.

## Nomenclature

AIP: atherogenic index of plasma
ALP: alkaline phosphatase
APOA1: Apolipoprotein A1
APOB: Apolipoprotein B
BMI: body mass index
BSA: body surface area
CAVD: calcific aortic valve disease
CT: computed tomography
Cys-c: Cystatin C
DBP: diastolic blood pressure
HbA1c: glycated hemoglobin A1c
HDL: high-density lipoprotein
LDH: lactate dehydrogenase
LDL: low-density lipoprotein
LY%: lymphocyte percentage
NEUT%: neutrophil percentage
NLR: neutrophil-to-lymphocyte ratio
SBP: systolic blood pressure
TC: total cholesterol
TG: triglyceride
UA: uric acid
WBC: white blood cell
